# The Relationship of Safety with Burnout for Mobile Health Employees

**DOI:** 10.3390/ijerph15071461

**Published:** 2018-07-11

**Authors:** Michael P. Leiter, Lois Jackson, Ivy Bourgeault, Sheri Price, Audrey Kruisselbrink, Pauline Gardiner Barber, Shiva Nourpanah

**Affiliations:** 1 School of Psychology, Burwood Campus, Deakin University, Geelong 3006, Australia; 2 School of Health and Human Performance, Dalhousie University, 6230 South Street, Halifax, NS B3H 3J5, Canada; Lois.Jackson@dal.ca; 3 Telfer School of Management, 55 Laurier Av. E. University of Ottawa, Ottawa, ON K1N 6N5, Canada; ivy.bourgeault@uottawa.ca; 4 School of Nursing, Room 122, Forrest Bldg., Dalhousie University 5869 University Avenue, Halifax, NS B3H 4R2, Canada; pricesl@dal.ca; 5 Centre for Organizational Research & Development, Box 220, Acadia University, Wolfville, Nova Scotia, NS B4P 2R6, Canada; Audrey.kruisselbrink@acadiau.ca; 6 Department of Sociology and Social Anthropology, Dalhousie University, Room 3112, McCain Building, 6135 University Avenue, Halifax, NS B3H 4R2, Canada; pgbarber@dal.ca; 7 Department of Sociology and Social Anthropology, Dalhousie University, Halifax, NS B3H 4R2, Canada; sh596189@dal.ca

**Keywords:** mobility, burnout, safety, healthcare, incivility, workload

## Abstract

Objective: The study examined the relationship of occupational safety with job burnout. Design: The study used a cross-sectional survey design. Setting: The setting was Nova Scotia, Canada. Participants: Mobile health employees (*N* = 156) completed surveys on road safety, workload, burnout and supervisor incivility. Main outcome measure: The main outcome measure was the Maslach Burnout Inventory. Results: Results found that safety concerns improved the prediction of exhaustion beyond that provided by workload concerns alone. Further, confidence in safety buffered the relationship of exhaustion with cynicism such that the exhaustion/cynicism relationship was stronger for employees who had lower confidence in road safety. Conclusions: Employees’ confidence in occupational safety while addressing work responsibilities on the road has implications for their experience of job burnout.

## 1. Introduction

Mobility is risky. Every form of transportation presents hazards. People get lost, experience discomfort, encounter difficult people and endure foul weather while moving from one place to another. Employment-related geographic mobility (ERGM), defined as “frequent and/or extended travel from places of permanent residence for the purpose of, and as part of, employment” [1], takes employees into unfamiliar terrain en route to employer-determined destinations on employer-determined schedules. With mobility as a pervasive quality of contemporary work [2,3], employees’ sense of safety on the road has implications for their engagement and satisfaction with their work and their wellbeing. The research reported here examines factors contributing to employees’ sense of safety both en route to work and during the workday, with reference to the implications of safety for burnout. Winter weather conditions comprise a major hazard for drivers in Canada, who experienced 160,315 road injuries in 2016, 1898 of which were fatal [4], with a greater rate of fatalities on rural roads. It is estimated that winter weather contributed to over 22% of these accidents [5]. These reports emphasize that driving in wintry conditions calls for distinct driving skills and specific equipment, such as snow tires. Whereas employers can play a role in developing employees’ skills and contributing to the relevant equipment, driving in winter conditions provides a useful focus for considering issues pertaining to safety concerns of employment-related geographical mobility.

## 2. Mobile Health Care

Employment projections in the developed world anticipate growth in the extent to which health care will be delivered in remote treatment settings and within service recipients’ homes [3,6]. These reports also note that many positions in this growing employment sector are modestly paid, receiving around $22,000 (U.S.) annually. The work is performed primarily by women whose caregiving roles, as well as the inherent mobility required to care for others receive little respect [7]. A modestly-paid position limits employees’ capacity to address shortcomings at work. For example, well-paid employees can improve their safety on the road by buying a better car, maintaining it more thoroughly and equipping it more effectively (e. g., winter tires, roadside assistance services). Poorly-paid employees lack that capacity. Within these constraints, mobile healthcare workers express concerns about managing mobility risks inherent in their work [8].

In the research setting for this study, Nova Scotia, Canada, many mobile employees use their personal vehicles to travel to work sites with costs reimbursed on a per-kilometer basis. Nova Scotia is largely rural with 920,000 people on 55,000 km^2^. In contrast, Belgium has 11.35 million people on 30,000 km^2^. The sparse population and modest economic status of Nova Scotia result in communities being only thinly served by public transportation.

Many studies identify mobility as a distinct risk for mobile healthcare providers [9,10]. A growing aspect of working life for healthcare providers in the contemporary world is mobility. The structure of mobile healthcare makes safety getting to and on the job a personal concern for employees. They must use more individual initiative to adapt their work practices to the diverse environments they inhabit in their workdays. One setting for mobile healthcare workers is within clients’ homes. Whether going to clients’ homes or to remote treatment settings, driving between locations in a personal automobile makes the employees responsible for transportation safety. The extent to which employees feel confident of their abilities to get to work or get between work places, which is a separate skill from actually doing the care work once they are at their designated workplace, and their employers’ support may play an important role in whether they experience burnout or engagement in their work.

## 3. Safety and Burnout

The history of occupational health psychology describes a shift from focusing exclusively on improving the actions of individual employees to appreciating the role of workplace context on safety outcomes. The contemporary ethos explores ways of integrating person and context into a comprehensive model for understanding and managing safety at work [10,11]. As noted, driving to work locations in a personal automobile creates an intersection between employee driving skills and employer policies on safety. Employee safety compliance, which is always less than perfect [8], could decrease when employees are both away from the workplace and in the context of their personal automobile. In a comprehensive meta-analysis across industries, Nahrgang. Morgeson and Hofmann [12] found support for a model in which job burnout, as a syndrome of exhaustion, cynicism and inefficacy [13], mediates the relationship of both demands and resources on safety behavior and safety outcomes. Explicit risks, as well as overall work demands contributed to exhaustion, while resource shortfalls made an additional contribution to burnout with implications for safety. Lack of support—both personal and managerial—was the most salient resource issue for this model. 

Cox and Tait [14] argued that employees assess the riskiness of workplace hazards as a function of the hazard’s capacity to harm and the hazard’s prevalence in the workplace. Similar dynamics may relate to employees’ travel to and from work sites. As emphasized in the transactional model of stress, control arising from employees’ capabilities and resources moderate the stressfulness of encounters with hazards [15]. The control component of safety appraisals integrates employees’ evaluation of their personal abilities with their evaluation of the employer’s support through training and materials. Although the hazards (prevalence and harmfulness) reflect the vagaries of weather and roadway events, the resources for addressing those hazards have more to do with the person and the workplace policies. In contrast to the vagaries of weather, employers’ policies on safety training or equipment reflect deliberate policy decisions with implications for employees’ wellbeing. The mobile health services sector has failed to articulate far-reaching policies in this regard in Canada.

Strains in the supervisory relationship play an integral role in employees’ experience of safety at work and their adherence to safe working practices [16]. The importance of the supervisory relationship goes beyond the issues of training and monitoring to encompass the social culture of a workgroup, including its support for maintaining a viable work/family interface [17]. A broadly-supportive supervisor relationship looks beyond the immediate job setting to reflect concern for employee wellbeing as realized in supervisory behaviors supporting a safe and fulfilling work life. The opposite of such a relationship is reflected in supervisor incivility characterized by exclusion, rude behavior and aspects of social undermining [18,19]. Supervisor incivility may have additional implications for employees’ confidence in managing workplace hazards.

In turn, employees’ feelings of safety at work have implications for both the exhaustion and cynicism aspects of burnout. First, addressing occupational hazards adds to workload arising from task demands: hazards are one more thing needing attention. Second, occupational hazards introduce uncertainty and anxiety that increase burdens on employees beyond those from demands [20]. Additionally, unsafe work environments and safety threats during work-related mobility discourage engagement, instead prompting employees to distance themselves both physically and psychologically [12]. Through these relationships, safety concerns have the potential to aggravate burnout.

In addition to these direct relationships of safety with burnout, safety has implications for the relationship of exhaustion with cynicism. Longitudinal analyses have demonstrated that when exhaustion and cynicism are inconsistent (one low, the other high), they tend to become consistent over time [21,22]. Moderating factors—fairness and information flow—play a role in whether they resolve in a positive direction (low on both) or in a negative direction (high on both).

These findings emphasize the importance of exploring factors that mediate or moderate the relationship of exhaustion with cynicism. The most straightforward process is that active involvement or dedication requires energy. Employees’ confidence in the safety of their work environment may moderate the extent to which those scoring high on exhaustion develop the full burnout syndrome [23].

## 4. Hypotheses

This analysis focuses on the direct and moderating relationships of safety with the cynicism aspect of the burnout syndrome. The first hypothesis concerns the direct path from safety to cynicism, predicting that employees who have confidence in their driving ability, have received training on managing wintery driving conditions and whose employers contribute to safety equipment will experience less cynicism. One proposed dynamic in this relationship is that employer support for safety conveys support for employee safety as a core value. In contrast, employees may experience low support for safety as unfair treatment that reduces their sense of control in their jobs when they must look after themselves on the road.

**Hypothesis** **1.** *Employees’ sense of safety will be negatively related to cynicism.*

**Hypothesis** **2.** *Employees’ sense of safety will be negatively related to exhaustion.*

**Hypothesis** **3.** *Strained supervisor relationships will negatively predict employees’ sense of safety and exhaustion.*

**Hypothesis** **4.** *The sense of safety will buffer the relationship of exhaustion with cynicism. Specifically, the relationship of exhaustion with cynicism will be stronger when safety is low than when safety is rated high.*

**Hypothesis** **5.** *The relationships described in Hypothesis 1–4 will occur in the context of the path from manageable workload to exhaustion to cynicism.*

In our hypothesized model, the well-established path from workload to exhaustion to cynicism provides the context for exploring the role of safety and supervisor relationships [24]. Although surveys consistently find correlations of workplace stressors and cynicism, analyses determine that exhaustion mediates these relationships [13]. Whereas the relationships of exhaustion with both workload and cynicism are fundamental to all models of burnout, it is necessary to build more elaborate models on the foundation of workload leading to exhaustion that in turn leads to cynicism. Considering the contribution of safety without also considering the role of workload presents a risk of mis-specifying the model. We propose the role of safety explored in this analysis to be in addition to what is already well established about burnout. Although some workplace strains, such as value conflicts and poor coworker relationships, have direct paths to cynicism, strains related to workload relate to cynicism to the extent that they are related to exhaustion [13]. Whereas supervisors have immediate authority over employees’ workload and the workgroup’s safety culture, the model proposes that exhaustion mediates the relationship of both workload and supervisor incivility with cynicism. The model further proposes exhaustion to mediate the relationship of safety with cynicism. This path acknowledges the variance shared between exhaustion and cynicism as reflecting a parallel process in which exhaustion relates immediately with external strains, while cynicism reflects to some degree employees’ level of exhaustion.

## 5. Method

### 5.1. Participants

Participants included 143 females and 13 males, of whom 120 held certificates for homecare work. All but one worked in healthcare, with 107 working in homecare, and that record was deleted. They included fulltime (93) part-time (24), casual (17) and temporary (1) employees (22 not indicating). Their average age was 41.57, ranging from 21–69. Participants were recruited through three organizations: the Nova Scotia Government & General Employees Union (NSGEU), the Nova Scotia College of Social Workers (NSSW) and graduates of the Nova Scotia Community College (NSCC). Education included college diplomas (108), bachelor’s degrees (20), high school diplomas (10) and post-bachelor degrees (18). Their average commute distance was 24.85 km (SD 22.60), requiring on average 24.07 min (SD 17.92). At work, they traveled an average of 12.08 days per month (SD 9.55) for a distance of 54.50 km (SD 57.75). Most (149) drove their own car to work, with the others carpooling (4), walking (3) or taking public transportation (1). Through a series of *t*-tests, we determined that the subsample comprising the NSGEU and NSSW participants did not differ (*p* < 0.05) on any of the measures in the study from the subsample of NSCC participants.

The disproportionately small number of men in the sample prohibits a meaningful analysis of differences between men and women. A series of *t*-tests found no significant differences between men and women (*p* < 0.05) on any of the measures in the study. A comparison of the correlation tables for the full sample and the sample comprising only women found no differences in the strength of correlations between any pair of variables. In light of these findings, the analysis proceeded on the full sample of healthcare employees.

### 5.2. Procedure

Upon receiving ethics approval from the participating universities and organizations, the NSGEU and NSSW offices sent a standard email inviting recipients to participate in the survey. The message included a link to the survey site. Six months later, the NSCC agreed to participate, as well, with their offices sending the message to graduates of Licensed Practical Nursing and Continuing Care Assistant programs. Reminders were sent in accordance with the approved research protocol with the surveys closing one month after opening, respectively.

### 5.3. Measures

Burnout: Burnout was measured with the 16-item Maslach Burnout Inventory—General Scale (MBI—GS) [25], assessing exhaustion, cynicism and efficacy. Participants used a 7-point frequency scale (ranging from 0-never to 6-daily) to indicate the extent to which they experienced each item (e.g., “I feel emotionally drained from my work”).

Incivility: Incivility was measured with the 12-item Straightforward Incivility Scale [26], which included three subscales: supervisor, coworker and instigated incivility.

Workload and resources: The Areas of Worklife Scale (AWS) [27] includes six scales on which participants indicate the extent to which their experience aligns with their expectations or aspirations for work, within six different areas: workload, control, reward, community, fairness and values.

Safety: Perception of safety was assessed with a questionnaire developed for this study that followed the general structure of a questionnaire developed for a study of hazards in the maintenance of military aircraft [28].

We asked respondents to rate the hazard of losing control of a car in bad weather. We asked them to rate:

- I feel at risk of injury from this hazard (riskiness),
- This hazard can produce a serious injury (severity),
- My skills and experience give me confidence in handing this hazard (control),
- I have received safety procedures training for dealing with this hazard (training),
- I have reimbursement for items to reduce my risk (e.g., snow tires, GPS) (resources).

### 5.4. Analysis

**Figure 1:**
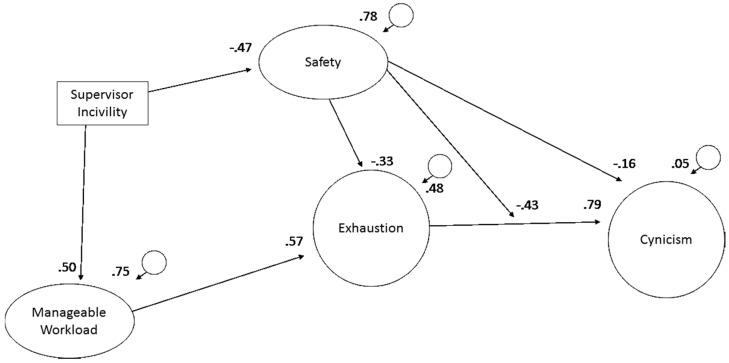
Safety moderation model with standardized coefficients. Note: all paths are statistically significant, *p* < 0.05.

The structural equation model (Figure 1) focuses on the central research questions, while acknowledging the limitations inherent in the modest sample size (*N* = 142). First, the model includes the path from workload to exhaustion and from exhaustion to cynicism, which are foundational features of job burnout models [13]. The model included supervisor incivility to reflect both the quality of the supervisory relationship and the overall civility of the workplace. The model includes the product of exhaustion and safety to test the buffering hypothesis. The alternative of a multi-level test contrasting subgroups with high and low levels of safety requires a much larger sample to provide sufficient power.

## 6. Results

### 6.1. Descriptive Statistics and Correlations

Table 1 displays the means, standard deviations and correlations for the variables in the study, in addition to the five variables in the structural model (exhaustion, cynicism, manageable workload, supervisor incivility and safety). The pattern of correlations among the burnout, areas of work life and incivility variables is consistent with other research [20,29]. Safety is significantly correlated with all of the other variables in the expected directions (positively with positively-framed constructs; negatively with negatively-framed constructs).

Distance was computed by multiplying the number of days travelled over the previous four weeks times the average kilometers travelled on a workday. Distance’s only significant (*p* > 0.05) correlation was *r* = −0.32 with coworker incivility. As noted, sex was not significantly related to any of the other variables. The only significant correlation for age was *r* = −0.17 with fairness, indicating older employees rated workplace justice less positively than did younger employees.

### 6.2. Structural Model

Confirmatory factor analysis: A confirmatory factor analysis (CFA) was conducted with EQS (EQuationS) [30]. In this analysis, the latent variables for exhaustion, cynicism, manageable workload, supervisor incivility and safety were each defined with three items. Because sex, age and distance travelled showed few relationships with the variables in the model, they were not included in the analysis. No error covariances were freed; the first item in each factor was fixed at 1.00, and the other items within each factor were freed. The sixth factor was the safety × exhaustion interaction term defined as a single item that was the product of the standardized scores for safety and exhaustion. Factors were free to correlate. The CFA supported a six-factor model with an excellent fit (*χ* ^2^_(89)_ = 106.95 *p* = 0.095; CFI (Comparative Fit Index) = 0.975, RMSEA = 0.042). In contrast, a five-factor model (combining exhaustion and cynicism into one factor) produced a significantly worse fit (*χ*^2^_(93)_ = 134.60 *p* = 0.003; CFI = 0.941, RMSEA = 0.063; difference Chi-square = 27.70, *p* < 0.001). This analysis confirms that the items form distinct factors as expected.

**Figure 2:**
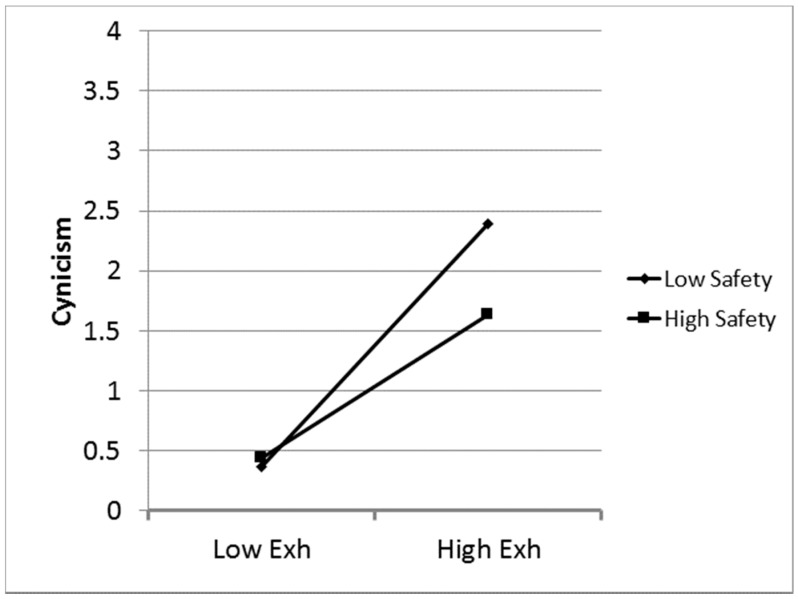
Buffering effect of safety on the relationship of exhaustion with cynicism.

Causal model: A structural equation analysis conducted with EQS [30] evaluated Hypotheses 1–5. An interaction term was defined as the product of the standardized scores of exhaustion and safety. All of the relationships depicted in Figure 2 are significant. The overall model fit is excellent (*χ* ^2^_(97)_ = 132.33, *p* = 0.133, CFI = 0.967, RMSR = 0.038). As noted in Table 2, the moderated model improved the fit over the same model without the moderator (*χ*^2^_(1)_ = 4.28, *p* < 0.05), which was a significant improvement over the structural null model that assigned items to factors without any structural paths among the factors (*χ* ^2^_(6)_ = 189.38, *p* < 0.01). Figure 1 depicts the relationships among the variables as specified in Hypotheses 1–5. Figure 2 displays a plot of safety’s moderation of the relationship of exhaustion with cynicism.

## 7. Discussion

The analysis demonstrated an active role for employees’ sense of safety within a model of job burnout in the context of mobile healthcare. First, employees’ feelings of safety on the job were negatively associated with supervisor incivility. Second, a path from safety to exhaustion enhanced the prediction of exhaustion by unmanageable workload in that safety explained variance in exhaustion beyond that explained by workload. Third, safety had a negative direct relationship with cynicism. Fourth, safety buffered the relationship of exhaustion with cynicism. These findings have implications for developing policies to assure safety during mobility for workers providing healthcare services to people remotely, a significant and growing part of the health workforce.

The association of supervisor incivility with safety points towards a potential point of intervention to improve employees’ quality of work life. Three of the five items on the supervisor incivility scale describe a distant working relationship, assessing how often supervisors ignored, excluded or behaved without consideration for the respondent. The other two items cite rude actions and words. Two of the five safety items fall within management’s domain: access to training and material support for safety measures. Certainly, rude words and behavior undermine working relationships, but the more direct quality for the relationship of supervisor incivility with safety may be distance, as reflected in encounters that leave employees feeling ignored and excluded.

From the supervisors’ perspective, lacking organizational policies that encourage and support providing employees with safety resources, supervisors may resort to actively avoiding the issue. That is, a distant relationship with employees may not reflect supervisors’ ill intent or personality, but reflect the bind they experience when lacking resources appropriate for their supervisory responsibilities. The quality of the supervisory relationship has been demonstrated to play a central role in nurses’ experience of their jobs and their profession. The results could be both better supervisory relationships, as well as safer work lives for employees.

Manageability of workload could be addressed by having employees empowered to problem solve necessary changes to workload throughout the day and communicate issues that arise. Furthermore, articulating clear scopes of practice that fully appreciate the competency of nurses would support a more proactive approach to work.

The attention given to the safety of employees providing health and support services in homes has highlighted the diverse challenges evident across cultures. Consistent with other Canadian researchers [10,31,32], our analysis found that winter driving conditions presented logistical, financial and psychological challenges for mobile workers. The autonomy implicit in working away from an organizational base [33] also presented risks inherent in working independently. Weather of course is not the sole concern of mobile workers. Mumtaz et al. [34] considered the hazard of men stalking women providers in Pakistan. Lang et al. [35] considered aggressive patients, unsanitary conditions and threatening animals in western Canada. It is clear that mobile employees face a diverse range of hazards with varying levels of prevalence and lethalness. A more thorough exploration of employees’ approaches to managing these risks would better inform employers’ responsibility for managing those risks. 

## 8. Conclusions

In terms of the workplace policy implications of the results, it suggest that employees would appreciate employers increasing their explicit support for mobility safety. The correlational results from this study argue for testing the connection more rigorously through research designs that would directly evaluate the impact of increased road safety support—both training and materials—on employees’ level of exhaustion and cynicism, and we note that policy action lags far behind recognition of safety concerns for mobile health providers.

## Figures and Tables

**Table 1:** Means, SD and correlations among the variables in the study.

Variable	Mean	StdDev	α	2	3	4	5	6	7	8	9	10	11	12	13	14	15
1 Exhaustion	2.36	1.50	0.92	0.73	−0.22	−0.47	−0.35	−0.29	−0.17	−0.35	−0.56	0.34	0.19	−0.35	−0.01 ‡	−0.03 ‡	−0.14 ‡
2 Cynicism	1.29	1.23		0.83	−0.38	−0.38	−0.28	−0.37	−0.19	−0.34	−0.67	0.43	0.15	−0.36	0.12 ‡	0.05 ‡	−0.13 ‡
3 Efficacy	4.31	0.98			0.87	0.14 ‡	0.21	0.30	0.24	0.23	0.36	−0.16	−0.27	0.39	0.12 ‡	−0.01 ‡	0.08 ‡
4 Workload	3.02	0.90				0.65	0.36	0.12	0.16	0.32	0.36	−0.26	−0.17	0.17	0.06 ‡	0.00 ‡	0.14 ‡
5 Control	3.40	0.83					0.87	0.30	0.24	0.43	0.40	−0.29	−0.06 ‡	0.26	0.01 ‡	−0.03 ‡	−0.09 ‡
6 Reward	3.48	0.81						0.81	0.66	0.34	0.38	−0.43	−0.28	0.32	−0.02 ‡	0.11 ‡	−0.10 ‡
7 Community	3.46	0.83							0.80	0.28	0.26	−0.22	−0.49	0.26	0.05 ‡	0.07 ‡	0.00 ‡
8 Fairness	2.89	0.73								0.65	0.52	−0.35	−0.20	0.25	−0.01 ‡	0.10 ‡	−0.17
9 Values	3.37	0.88									0.77	−0.50	−0.14 ‡	0.36	0.03 ‡	0.08 ‡	0.03 ‡
10 Supervisor Incivility	0.71	1.10										0.87	0.24	−0.28	0.11 ‡	−0.02 ‡	0.14 ‡
11 Coworker Incivility	0.58	0.79											0.94	−0.17	−0.32	−0.11 ‡	−0.02 ‡
12 Safety	2.64	0.82												0.65	−0.02 ‡	0.14 ‡	0.08 ‡
13 Distance *	792.43	864.56													n/a	0.04 ‡	0.02 ‡
14 Sex	1.09	0.28														n/a	−0.06 ‡
15 Age	42.42	11.26															n/a

Note: *N* = 142, all correlations significant *p* < 0.01, except for ‡; Cronbach’s alpha on the main diagonal. Sex: 1 = female; 2 = male. Distance: days traveled over the past 4 weeks * average km driven per workday.

**Table 2:** Goodness of fit for the hypothesized model.

Model	*χ* ^2^	df	Sig	CFI	RMSR	*χ*^2^ Difference
Null Model	827.40	114	0.001	0.050	0.186	
Structural Null Model	325.99	104	0.001	0.662	0.116	501.41
No Moderator Model	136.61	98	0.113	0.963	0.039	189.38
Moderated Model	132.33	97	0.133	0.967	0.038	4.28
